# Botulism

**DOI:** 10.21980/J8FD0R

**Published:** 2020-01-15

**Authors:** John Thompson, Zane Horowitz, Adam Blumenberg

**Affiliations:** * Oregon Health and Science University, Department of Emergency Medicine, Portland, OR; ^ Columbia University Medical Center, Department of Emergency Medicine, New York City, NY

## Abstract

**Audience:**

This simulation is targeted to emergency medicine residents and medical students. This case focuses on the diagnosis and management of botulism toxicity, while highlighting the logistical complications of botulism toxicity.

**Introduction:**

Botulism is a potentially life-threatening emergency that often presents with subtle symptoms, which can progress to paralysis and respiratory failure. A descending flaccid paralysis is typical, initially affecting smaller muscles such as oculomotor, then larger facial muscles. 1,2 Early indications of respiratory compromise are important to recognize. It is important for emergency medicine physicians to be familiar with botulism and recognize the presentation quickly to safely treat the patient. Clinical findings may include: dilated pupils, diplopia, xerostomia, dysphagia, and nausea and vomiting. 3 Treatment priorities include assessment and management of the airway, close monitoring, and coordinating with local agencies to obtain botulinum antitoxin.^1^

**Educational Objectives:**

By the end of this simulation learners will be able to: 1) develop a differential for descending paralysis and recognize the signs and symptoms of botulism; 2) understand the importance of consulting public health authorities to obtain botulinum antitoxin in a timely fashion; 3) recognize that botulism will progress during the time period antitoxin is obtained. Early indications of respiratory compromise are expected to worsen during this time window.

Secondary learning objectives include: 4) employ advanced evaluation for neurogenic respiratory failure such as physical examination, negative inspiratory force (NIF), forced vital capacity (FVC), and partial pressure of carbon dioxide (pCO2), 5) discuss and review the pathophysiology of botulism, 6) discuss the epidemiology of botulism.

**Educational Methods:**

This simulation was conducted using a high-fidelity mannequin with intubating capabilities and real-time vital sign monitoring. Following the simulation, the participants underwent a debriefing session and discussion on botulism. This case was designed as a high-fidelity simulation, but it can be adapted to a low-fidelity simulation or case discussion.

**Research Methods:**

Following the simulation and debriefing session, participants were provided with a survey to rate the simulation and provide feedback to instructors. Participants were asked open-ended questions about the strengths and areas of improvement of the case, and were asked to rate how they valued the learning content of the case on a 5-point scale.

**Results:**

Emergency medicine residents expressed positive feedback on the scenario. The residents appreciated the change in clinical course of the patient over time as well as the presentation of botulism.

**Discussion**

This simulation is an effective way of teaching about botulism to emergency medicine residents. We used a primary nurse asking questions to progress the case and stimulate the learners to think about certain specific aspects of the case, such as the patient’s weakness or disposition.

**Topics**

Toxicology, botulism, emergency medicine, medical simulation.

## USER GUIDE

**Table:** 

List of Resources:	
Abstract	1
User Guide	3
Instructor Materials	5
Operator Materials	15
Debriefing and Evaluation Pearls	17
Simulation Assessment	19

**Learner Audience:**

Medical students, interns, junior residents, senior residents

**Time Required for Implementation:**

Instructor Preparation: 30 minutes

Time for case: 15 minutes

Time for debriefing: 40 minutes

**Recommended Number of Learners per Instructor:**

3–4

**Topics:**

Toxicology, botulism, emergency medicine, medical simulation.

**Objectives:**

By the end of this simulation learners will be able to:

1. Develop a differential for descending paralysis and recognize the signs and symptoms of botulism.
2. Understand the importance of consulting public health authorities to obtain botulinum antitoxin in a timely fashion.
3. Recognize that botulism will progress during the time period antitoxin is obtained.

Secondary learning objectives include:

4. Employ advanced evaluation for neurogenic respiratory failure such as physical examination, negative inspiratory force (NIF), forced vital capacity (FVC), and partial pressure of carbon dioxide (pCO2).
5. Discuss and review the pathophysiology of botulism.
6. Discuss the epidemiology of botulism.

### Linked objectives and methods

Botulism is an uncommon emergency department (ED) presentation with subtle nonspecific symptoms. Other diagnoses that could be considered include but are not limited to: tick paralysis, myasthenia gravis, cerebrovascular accident, multiple sclerosis, malignancy, and ciguatera toxicity. This scenario emphasizes the importance of maintaining a high clinical suspicion for botulism in at risk populations, which can be ascertained from thorough history gathering (objective 1). Learners will need to identify early signs of respiratory failure through thorough examination and ancillary testing (objective 4). Once diagnosed, learners will need to facilitate obtaining the antitoxin and consult the appropriate services/agencies (objective 2). While waiting for the antitoxin to arrive, learners will need to reassess the patient and continue to monitor their respiratory status and disposition appropriately (objective 3). Following the simulation there will be a debriefing and discussion on the etiology, epidemiology, and pathophysiology of botulism (objectives 5,6). This simulation will reinforce the prompt evaluation, diagnosis, management, and reassessment necessary to appropriately treat botulism (objectives 1–6) in a safe learning environment, and participants will receive feedback on their performance.

### Recommended pre-reading for instructor

If the instructor is not familiar with botulism, we recommend any of our textbooks referenced in the references section, such as *Goldfrank’s Toxicologic Emergencies*. Reviewing the local policies (if in place) in their hospital for obtaining antitoxin may be beneficial to customize the simulation to the local environment of the learners.

### Results and tips for successful implementation

This simulation was designed for emergency medicine residents on a toxicology rotation. It was performed in a high-fidelity simulation setting; however, it can be adapted to be an oral case discussion or performed in a low-fidelity scenario. This case was designed and implemented during the 2019–2020 academic year. The case was piloted with 12 learners over three sessions spaced one month apart each. All learners were from the same institution, and there are typically 3–4 learners per month. Learners were queried in person and via anonymized online survey about the strengths and potential improvements of the case. All respondents indicated 5 out of 5 (maximal agreement) to the following three statements:

- This experience will improve my performance in actual clinic setting.
- This simulation was a valuable learning experience.
- The debriefing was a valuable learning experience.

Thematic analysis of the two open-ended questions “How could this experience be improved?” and “What were the strengths of this experience?” were universally positive. Specifically, learners appreciated the differential diagnosis, diagnostic approach to the rare case, the discussion of the approach to obtaining antitoxin, and how the case required a thorough physical examination and performance of a social history. Iterative improvements were made to the case (more detailed differential diagnosis, more detailed neurological examination, and addition of cueing prompts by nurse when necessary) based on feedback from learners and administrators, resulting in its present formulation.

To provide help during the simulation to the learners if necessary, the primary nurse would ask questions about the patient periodically to guide them to think about respiratory status and disposition. The learners appreciated the change in clinical course of the patient over time as well as the presentation of botulism. Based on anonymized surveys, learners valued a thorough differential in the debrief, the workup and management of suspected botulism, and the process to obtain the antitoxin.

## Supplementary Information



## Figures and Tables

**Table:** 

#1	Complete blood count (CBC)
#2	Basic metabolic panel (BMP)
#3	Liver function tests
#4	Coagulation studies
#5	Creatine phosphokinase (CPK)
#6	Toxin Screen Panel
#7	Arterial blood gas (ABG)
#8	Urinalysis
#9	Negative inspiratory force (NIF)
#10	Forced vital capacity (FVC)
#11	Chest Radiograph (CXR)
#12	Electrocardiogram (ECG)

**Table:** 

**Age:** 35	**Sex:** Male	**Method of transportation:** Walked

**Table:** 

**Vitals:**	*Heart Rate (HR)* 91	*Blood Pressure (BP)* 135/90	*Respiratory Rate (RR)* 16
	*Temperature (T)* 98.9°F axillary	*Oxygen Saturation (O_2_Sat)* 99% on room air	

**Table:** 

*Complete blood count (CBC)*	
White blood count (WBC)	8.0 x1000/mm^3^(H)
Hemoglobin (Hgb)	14.5 g/dL
Hematocrit (HCT)	42.0%
Platelet (Plt)	215 x1000/mm^3^
Segs:	80%
Lymphs:	9%
Monos:	7%
Eos:	4%

**Table:** 

*Basic metabolic panel (BMP)*	
Sodium	142 mEq/L
Chloride	106 mEq/L
Potassium	4.2 mEq/L
Bicarbonate (HCO_3_)	24 mEq/L (L)
Blood Urea Nitrogen (BUN)	12 mg/dL (H)
Creatine (Cr)	1.0 mg/dL (H)
Glucose	100 mg/dL

**Table:** 

*Liver Function Tests (LFTs)*	
Total bilirubin	0.8 mg/dL
Albumin	4.0 g/dL
Alkaline Phosphate	60 U/L
Total Protein	7.9 g/dL
Aspartate Aminotransferase (AST)	45 u/L
Alanine Aminotransferase (ALT)	37 u/L

**Table:** 

*Coagulation Studies*	
International Normalized Ratio (INR)	1.0
Partial Thromboplastin Time (PTT)	32 seconds

**Table:** 

*Creatine phosphokinase (CPK)*	88 U/L

**Table:** 

*Toxin Screen Panel*	
Acetaminophen	negative
Salicylate	negative
Ethanol	negative
Toxic Alcohol Panel	negative

**Table:** 

*Urine Toxicology Screen*	
Amphetamines	negative
Benzodiazepines	negative
Opiates	positive
Cocaine	negative
Tricyclics	negative
THC	negative

**Table:** 

*Arterial Blood Gas (ABG)*	
pH	7.32
pCO_2_	50 mmHg
PO_2_	70 mmHg
HCO_3_	22 mEq/L
O_2_ saturation	94%
Lactic Acid	1.5 mmol/L

**Table:** 

*Urinalysis (UA)*	
Color	yellow
Specific gravity	1.28
Glucose	negative
Protein	negative
Ketones	negative
Leukocyte esterase	negative
Nitrites	negative
White blood cells (WBC)	0–5 WBCs/high powered field (HPF)
Red blood cells (RBC)	0–5 RBCs/HPF
Squamous epithelial cells	0–5 cells/HPF

**Table:** 

*Urinalysis (UA)*	
Color	yellow
Specific gravity	1.28
Glucose	negative
Protein	negative
Ketones	negative
Leukocyte esterase	negative
Nitrites	negative
White blood cells (WBC)	0–5 WBCs/high powered field (HPF)
Red blood cells (RBC)	0–5 RBCs/HPF
Squamous epithelial cells	0–5 cells/HPF

**Table:** 

*Negative inspiratory force (NIF)*	18 cm H20

**Table:** 

*Forced vital capacity*	190 mL

**Figure:**
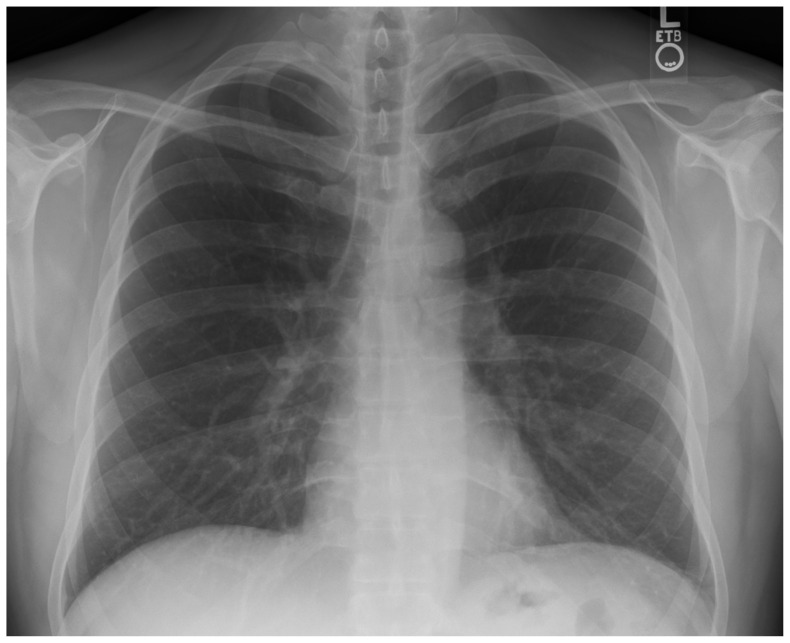


**Figure:**
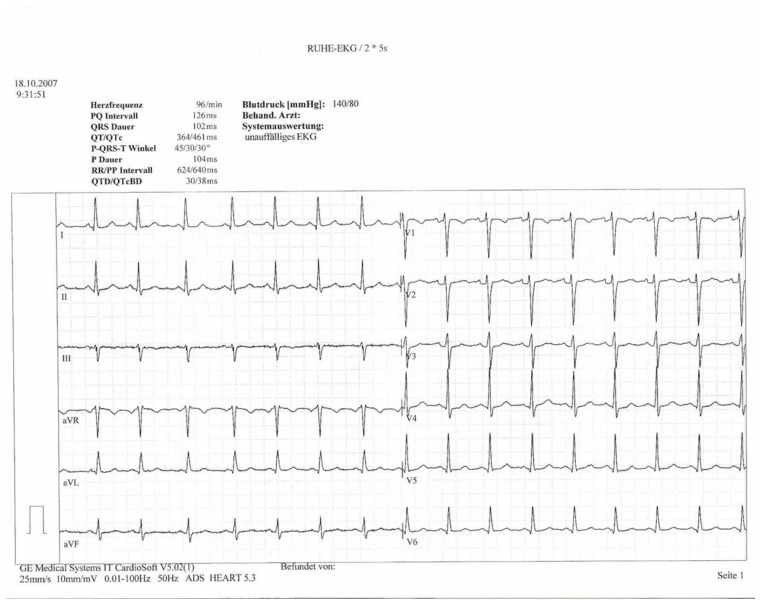


**Table:** 

Minute (state)	Participant action/trigger	Patient status (simulator response) & operator prompts	Monitor display (vital signs)
0:00 (Baseline)	Patient brought from waiting room to room	Participants should perform an ABC assessment, place patient on monitor, and perform a history and physical examination.	T: 98.9°F HR: 95 BP: 138/76 RR: 22 O2: 94%
04:00	IV placed, labs drawn, CXR ordered	Participants should perform a detailed neurological exam. If participants do not perform neuro exam, nursing will prompt with “Why is the patient laying so still?”	T: 98.9°F HR: 95 BP: 138/76 RR: 22 O2: 94%
	Lab results return	Participants should note the labs indicate a mild respiratory acidosis and are otherwise grossly normal.	T: 98.9°F HR: 95 BP: 138/76 RR: 22 O2: 94%
10:00	Participants should recognize respiratory muscle weakness.	If the team intubates the patient, he will stabilize. If the patient is not intubated, he will develop worsening respiratory function. If the patient is not pre-oxygenated prior to intubation, he will have an oxygen desaturation during the procedure. If the team does not intubate the patient, he will require intubation after admission, or suffer a cardiac arrest if not admitted to ICU. This must be clarified during the debrief.	Intubated: T: 99.8°F HR: 94 BP: 131/84 RR: 20 O2sat: 100% Not intubated: T: 98.9°F HR: 95 BP: 138/76 RR: 22O2: 94%
12:00	Participants should recognize the need for antitoxin	If the team contacts a public health agency the patient will receive antitoxin within 8 hours. If the team does not contact a public health agency the patient will not receive antitoxin and will have a prolonged illness. This must be clarified during the debrief.	

**Table:** 

**Critical Actions** Recognition of botulism poisoning Evaluation of respiratory function (clinically and with NIF/FVC) Intubate the patient Contact a public health agency for antitoxin Admission to ICU	0:00

**Table:** 

	Milestone	Did not achieve level 1	Level 1	Level 2	Level 3
1	**Emergency Stabilization (PC1)**	□ Did not achieve Level 1	□ Recognizes abnormal vital signs	□ Recognizes an unstable patient, requiring intervention Performs primary assessment Discerns data to formulate a diagnostic impression/plan	□ Manages and prioritizes critical actions in a critically ill patient Reassesses after implementing a stabilizing intervention
2	**Performance of focused history and physical (PC2)**	□ Did not achieve Level 1	□ Performs a reliable, comprehensive history and physical exam	□ Performs and communicates a focused history and physical exam based on chief complaint and urgent issues	□ Prioritizes essential components of history and physical exam given dynamic circumstances
3	**Diagnostic studies (PC3)**	□ Did not achieve Level 1	□ Determines the necessity of diagnostic studies	□ Orders appropriate diagnostic studies. Performs appropriate bedside diagnostic studies/procedures	□ Prioritizes essential testing Interprets results of diagnostic studies Reviews risks, benefits, contraindications, and alternatives to a diagnostic study or procedure
4	**Diagnosis (PC4)**	□ Did not achieve Level 1	□ Considers a list of potential diagnoses	□ Considers an appropriate list of potential diagnosis May or may not make correct diagnosis	□ Makes the appropriate diagnosis Considers other potential diagnoses, avoiding premature closure
5	**Pharmacotherapy (PC5)**	□ Did not achieve Level 1	□ Asks patient for drug allergies	□ Selects an medication for therapeutic intervention, consider potential adverse effects	□ Selects the most appropriate medication and understands mechanism of action, effect, and potential side effects Considers and recognizes drug-drug interactions
6	**Observation and reassessment (PC6)**	□ Did not achieve Level 1	□ Reevaluates patient at least one time during case	□ Reevaluates patient after most therapeutic interventions	□ Consistently evaluates the effectiveness of therapies at appropriate intervals
7	**Disposition (PC7)**	□ Did not achieve Level 1	□ Appropriately selects whether to admit or discharge the patient	□ Appropriately selects whether to admit or discharge Involves the expertise of some of the appropriate specialists	□ Educates the patient appropriately about their disposition Assigns patient to an appropriate level of care (ICU/Tele/Floor) Involves expertise of all appropriate specialists
9	**General Approach to Procedures (PC9)**	□ Did not achieve Level 1	□ Identifies pertinent anatomy and physiology for a procedure Uses appropriate Universal Precautions	□ Obtains informed consent Knows indications, contraindications, anatomic landmarks, equipment, anesthetic and procedural technique, and potential complications for common ED procedures	□ Determines a back-up strategy if initial attempts are unsuccessful Correctly interprets results of diagnostic procedure
20	**Professional Values (PROF1)**	□ Did not achieve Level 1	□ Demonstrates caring, honest behavior	□ Exhibits compassion, respect, sensitivity and responsiveness	□ Develops alternative care plans when patients’ personal beliefs and decisions preclude standard care
22	**Patient centered communication (ICS1)**	□ Did not achieve level 1	□ Establishes rapport and demonstrates empathy to patient (and family) Listens effectively	□ Elicits patient’s reason for seeking health care	□ Manages patient expectations in a manner that minimizes potential for stress, conflict, and misunderstanding. Effectively communicates with vulnerable populations, (at risk patients and families)
23	**Team management (ICS2)**	□ Did not achieve level 1	□ Recognizes other members of the patient care team during case (nurse, techs)	□ Communicates pertinent information to other healthcare colleagues	□ Communicates a clear, succinct, and appropriate handoff with specialists and other colleagues Communicates effectively with ancillary staff

## INSTRUCTOR MATERIALS

**Case Title:** Botulism

**Case Description & Diagnosis (short synopsis):** Patient is a 35-year-old male with history of black tar heroin use that presents to the emergency department with complaint of “my cigarettes keep falling out of my mouth.” He has been triaged as a low acuity patient and in the waiting room for four hours. He initially has normal vital signs; however, over the course of the simulation develops descending paralysis and respiratory failure. He will require thorough evaluation for neurogenic respiratory compromise with negative inspiratory force (NIF) and forced vital capacity (FVC) testing. Examination will reveal development of tachypnea, xerostomia, and peripheral weakness on neurological exam. Diagnostics will reveal hypoxia on room air and pending respiratory failure. He subsequently will require intubation and intensive care unit (ICU) admission. He will also require that the provider contact a public health authority such as their local poison center, state health department, or Center for Disease Control and Prevention (CDC) to arrange for delivery of botulinum antitoxin.

**Equipment or Props Needed:**

High fidelity simulation mannequin
Vital signs monitor
Pulse oximetry
Angiocaths for peripheral intravenous access (eg, 18 gauge, 20 gauge, 22 gauge)
Nasal cannula, non-rebreather mask
Intubating equipment
Simulated medications for intubation with labeling
Simulated antitoxin with labeling
Negative inspiratory force meter

**Confederates needed:**

Primary nurse

**Stimulus Inventory:**

**Background and brief information:** A 35-year-old man with a history of intravenous black tar heroin abuse presents to the Emergency Department (ED) for evaluation of dry mouth, weakness, and difficulty holding a cigarette in his mouth. He has been triaged as a low acuity patient and has been in the waiting room for four hours.

**Initial presentation:** This is the case of a 35-year-old man with history of daily intravenous heroin abuse who has developed wound botulism. Over the course of a few hours, he has descending paralysis which progresses to respiratory compromise and ultimately respiratory failure. The case will test the examinee’s ability to diagnose botulism, obtain the antitoxin, recognize abnormal vital signs and protect the patient from lethal respiratory paralysis.

**How the scenario unfolds:**

1. The patient will wait in the waiting room for 4 hours prior to physician evaluation.
2. The patient will have developed rapid shallow breathing (RR 22) and mild hypoxemia (94% on RA) during his time in the waiting room.
3. Physical examination will show symmetric abnormal lower motor neuron findings without sensory findings.
4. Patient will require botulinum antitoxin, which must be acquired by contacting a public health agency. It is expected that there will be a delay of 6–12 hours until the antitoxin reaches the patient. Antitoxin halts progression of paralysis but does not reverse it.
5. Patient is expected to develop progressive respiratory paralysis over the next several hours. Based on clinical evaluation, including possibly NIF and FVC, patient should be intubated.
6. Patient should be admitted to the ICU.

**Critical Actions:**

1. **Recognition of botulism poisoning**
  The patient will have cranial nerve weakness with progressive descending paralysis. he differential includes but is not limited to: botulism, myasthenia gravis crisis, Guillain-Barre syndrome, profound electrolyte disturbance (periodic hypokalemia), Parkinsonism, and tick paralysis. Botulism should be the leading diagnosis on history and physical examination.
  - Cuing Guideline: If the learners appear to be struggling with diagnosing the patient, a nurse can ask doctor why patient is lying so still.
2. **Place patient on monitor and obtain repeat vitals and obtain IV access**
3. **Evaluate respiratory function clinically or with NIF/FVC**
  The patient will have developed tachypnea (22 breaths per minute), shallow respirations, and hypoxemia (94% on room air) in the time he spent in the waiting room. Pending respiratory failure should be suspected, and the team should consider assessing NIF and FVC. This determination can be made without NIF/FVC with a thorough physical examination including complete neurologic exam and attention to the rapid progression of symptoms.
  - Cuing Guideline: Nurse can ask doctor if naloxone should be given for patient’s respiratory issues.
4. **Place patient on supplemental oxygen**
5. **Intubate the patient in the emergency department**
  Given the progression of respiratory weakness during the patient’s ED stay and the inherent delay in antitoxin acquisition, the patient should receive intubation in the ED. The patient will not develop respiratory failure in the simulation if intubated. Without intubation he is likely to develop respiratory failure over the next several hours and will code once admitted.
  - Cuing Guideline: Nurse can ask doctor how quickly botulism acts.
6. **Call public health agency to coordinate antitoxin delivery**
  In the United States, there are few hospitals that have botulism antitoxin readily available. Calling a public health agency (such as the poison center, state public health department, or CDC) is crucial to obtaining the antitoxin.
  - Cuing Guideline: Pharmacist or nurse can tell doctor the hospital has no antitoxin available and asks about the next step.
7. **Admission to the ICU**
  This patient will need ICU admission. Any attempt to admit elsewhere will meet minimal resistance by accepting physician. If admitted to the ward, the patient will develop unwitnessed respiratory failure and die overnight.
  - Cuing Guideline: Nurse can ask doctor about patient’s definitive disposition.

**Case Title:** Botulism

**Chief Complaint:** weakness, dry mouth, inability to hold cigarette in his mouth

**General Appearance:** Laying flat on stretcher, appears uncomfortable, not moving very much

**Primary Survey:**

- **Airway:** protecting airway
- **Breathing:** clear to auscultation bilaterally
- **Circulation:** distal intact pulses

**History:**

- **History of present illness**: Since this morning patient cannot hold a cigarette in his mouth and his mouth and throat feel dry. He noticed these symptoms this morning after waking up. Pertinent negatives: no fever or difficulty eating or swallowing, no sick contacts, no new medications.
- **Past medical history:** Asthma
- **Past surgical history:** None
- **Patient’s medications:** None
- **Allergies:** No known drug allergies
- **Social history:** Lives alone locally, unemployed. Drinks alcohol occasionally. Smokes marijuana, endorses intravenous black tar heroin abuse
- **Family history:** non-contributory

**Secondary Survey/Physical Examination:**

- **General appearance:** laying flat on stretcher, appears uncomfortable, not moving very much
- **Head, ears, eyes, nose and throat (HEENT):**
  ○ **Head:** WNL (within normal limits)
  ○ **Eyes:** pupils 3mm to 2mm with light. Ptosis noted if asked
  ○ **Ears:** TMs clear
  ○ **Nose:** WNL
  ○ **Throat:** dry oral mucosa when asked.
- **Neck:** supple
- **Heart:** Regular rate, distal pulses present. No M/R/G (murmurs, rubs, gallops)
- **Lungs:** clear symmetric breath sounds. Tachypneic, shallow respirations (provide if asked).
- **Abdominal/GI:** WNL
- **Genitourinary:** WNL
- **Rectal:** WNL
- **Extremities:** Full range of motion, no evidence of recent trauma
- **Back:** WNL
- **Neuro: (must ask for specific in-depth exam findings and perform neurological exam)**
  Speech intact, coordination intact, sensation intact, down going plantar reflex, ptosis, decreased facial muscle tone, bilateral pronator drift, 4/5 strength in all muscle groups, 1+ reflexes, falls back onto stretcher when he attempts to rise
  CN II: No visual field cut, 20/30 vision bilaterally
  CN III, IV, VI: Bilateral ptosis, unable to sustain lateral gaze
  CN V: Sensation in V1-V3 intact
  CN VII: Subtle bilateral facial droop, unable to maintain puffed-out cheeks
  CN VIII: Hearing intact
  CN IX: Uvula midline, normal palate rise
  CN X: Intact gag reflex, speech intact with soft voice
  CN XI: Shoulder shrug intact
  CX XII: No tongue deviation
- **Skin:** Needs to verbalize looking for skin findings related to IV drug use. Track marks on arms. Scabbed area on left antecubitum without fluctuance or erythema.
- **Lymph:** WNL
- **Psych:** WNL

**Results:**

*Chest X-ray:* Normal

Stillwaterising. Chest Xray PA. In: Wikimedia Commons.

https://commons.wikimedia.org/wiki/File:Chest_Xray_PA_3-8-2010.png. Published March 8, 2010. Public domain.

*ECG:*

A Normal twelve channel ECG. In: Wikimedia Commons.

https://commons.wikimedia.org/wiki/File:Ekg_normal_bionerd.jpg. Published October 18, 2007. CC BY 3.0.

## OPERATOR MATERIALS

**SIMULATION EVENTS TABLE:**

**Diagnosis:**

Botulism

**Disposition:**

Admit to the intensive care unit

## DEBRIEFING AND EVALUATION PEARLS

### Botulism

**Pearls:** Botulism is a clinical syndrome of progressive neuromuscular paralysis caused by the botulinum toxin. Botulinum is produced by the gram-positive bacteria *Clostridium botulinum*, as well as *C. butyricum* and *C. baratii*. Botulism may be transmitted in the following ways: Foodborne botulism occurs by pre-formed toxin in food such as improperly canned food or honey. Infantile botulism is caused by *C. botulinum* colonizing the immature intestinal tract and secreting toxin. Iatrogenic botulism is caused by an overdose of injected botulinum used in medical procedures. Wound botulism occurs when wounds become infected by *C. botulinum* and secrete toxin. It is most common among those who inject drugs intravenously.

The differential diagnosis for patients presenting with neuromuscular paralysis includes tick paralysis, myasthenia gravis, cerebrovascular accident, multiple sclerosis, hypermagnesemia, hypokalemia, Guillain Barre syndrome, and ciguatera toxicity.

#### Signs and Symptoms

1. Botulism causes a symmetric lower motor neuron paresis, typically starting with the cranial nerves and descending down the body. Reflexes are diminished and there should be no upper motor neuron findings such as spasticity, hyperreflexia, or increased tone. Initial cranial nerve findings such as ptosis and facial muscle flaccidity are typical. The signs of symptoms of botulism may be remembered by the "Dozen Ds" in order of their manifestation: Dry mouth, Diplopia, Dilated pupils, Droopy eyes (ptosis), Droopy face, Diminished gag reflex, Dysphagia, Dysarthria, Dysphonia, Difficulty lifting head, Descending paralysis, Diaphragmatic paralysis.
2. Botulism does not cause any sensory neuron dysfunction, pain, or altered mental status. Presence of these findings should prompt consideration of alternative diagnosis.
3. Patients often complain of dry mouth and throat.
4. Infantile botulism often presents with constipation.
5. If unchecked, patients become completely paralyzed and die from acute respiratory failure.

**Other debriefing points:** The antitoxin requires release from CDC after an emergency contact by a public health agency; due to administrative and transportation delays, this process may take several hours. Antitoxin prevents botulism from progressing, but does not reverse the neuromuscular paralysis that has already occurred. Therefore, it is crucial to consider progression of disease while the patent waits for antitoxin delivery, and to account for delays related to obtaining emergency release of medication from a government agency. Early intubation and mechanical ventilation should be considered if there will be a significant delay until antitoxin administration. The 24/7 phone number for obtaining botulinum antitoxin from the CDC is (770) 488 – 7100. This can also be coordinated by calling Poison Control at 1 – (800) 222 – 1222.

Wound botulism is often related to seemingly minor wounds that do not require interventions such as antibiotics or excision. Black tar heroin use has become a leading risk for development of wound botulism. If a patient has an infected wound it should be treated and debrided.

The epidemiology of botulism is primarily based on exposure history. Exposures to botulism include:

- Homemade preserved food (canned goods, “muktuk” which is traditional fermented whale blubber, “pruno” which is fermented alcohol product brewed in prison, viscous inadequately refrigerated food such as gas station cheese dip)
- Honey in neonates
- Black tar heroin use (more commonly seen in Western than Eastern United States)
- Iatrogenic sources (cosmetic or therapeutic botulinum injections)

**Wrap Up:** Botulinum disrupts the presynaptic SNARE proteins in the lower motor neurons. SNARE proteins are involved in release of acetylcholine vesicles. As a result, no acetylcholine can be released into the neuromuscular junction and paralysis ensues. The affected SNARE proteins are permanently disabled, and so restoration of neuron function requires synthesis of new SNARE proteins and axonal transport to the synapse. It may take weeks for neuron function to return. Botulinum antitoxin halts disease progression, but does not reverse it. For example, administering antitoxin may prevent a patient from requiring intubation if given early; however, patients treated with antitoxin will still require weeks to regain their strength and may require physical therapy, home health services, and hospital admission.

## SIMULATION ASSESSMENT

*Botulism*

Learner: _________________________________________

***Assessment Timeline***

This timeline is to help observers assess their learners. It allows observer to make notes on when learners performed various tasks, which can help guide debriefing discussion.

**Critical Actions:**

□ Recognition of botulism poisoning
□ Evaluation of respiratory function (clinically and with NIF/FVC)
□ Intubate the patient
□ Contact a public health agency for antitoxin
□ Admission to ICU

**Summative and formative comments:**

**Milestones assessment:**
