# Supplementary material for: Botulism
Source: J Educ Teach Emerg Med. 2020 Jan 15;6(1):S1–S23. doi: 10.21980/J8FD0R (PMC10332761; doi:10.21980/J8FD0R)
Supplement: Supplementary file 1 [file jetem-6-1-s1-supp1.pptx]

## Slide 1
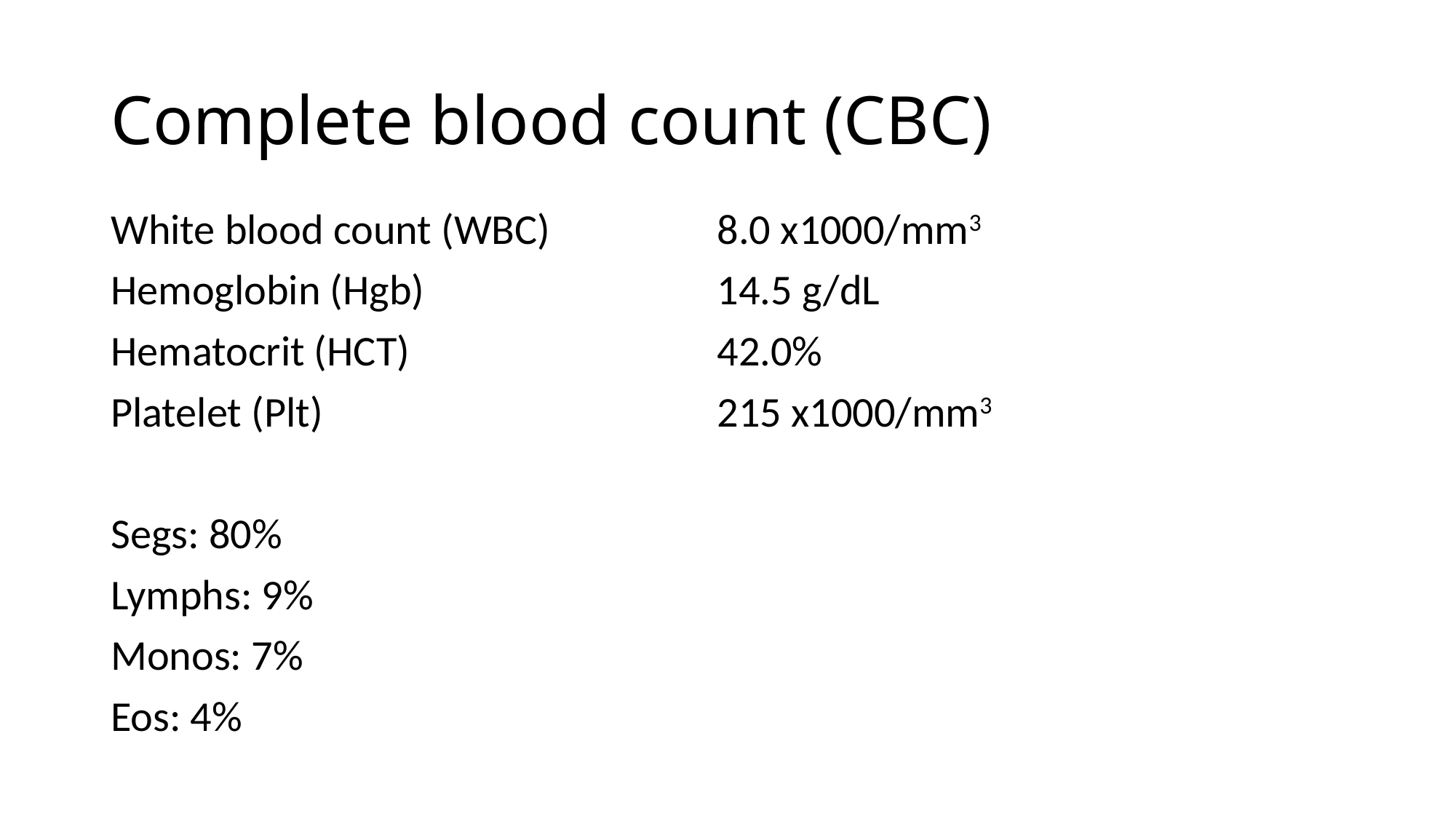

# Complete blood count (CBC)
White blood count (WBC)
Hemoglobin (Hgb)
Hematocrit (HCT)
Platelet (Plt)
Segs: 80%
Lymphs: 9%
Monos: 7%
Eos: 4%
8.0 x1000/mm3
14.5 g/dL
42.0%
215 x1000/mm3

## Slide 2
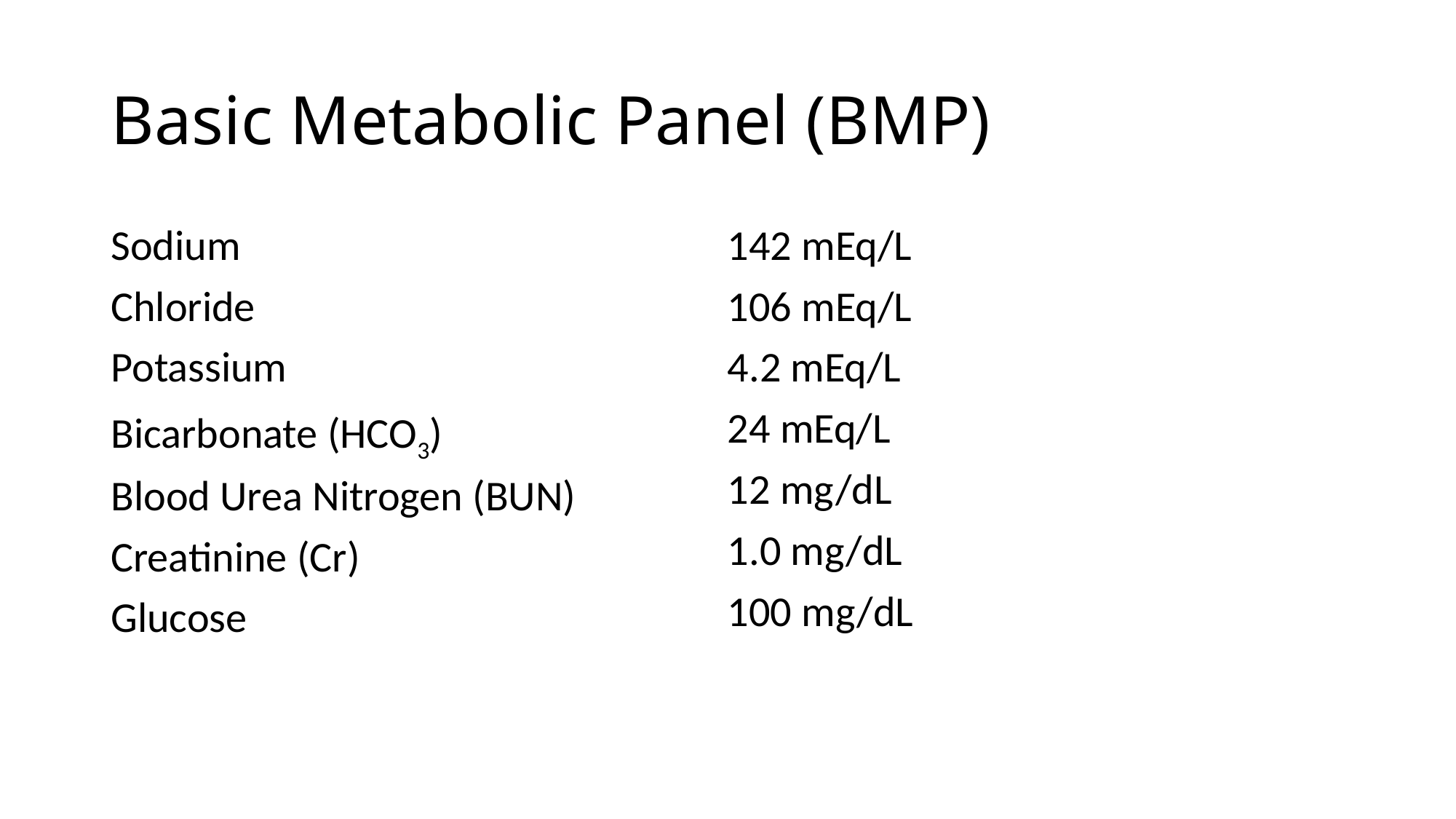

# Basic Metabolic Panel (BMP)
Sodium
Chloride
Potassium
Bicarbonate (HCO3)
Blood Urea Nitrogen (BUN)
Creatinine (Cr)
Glucose
142 mEq/L
106 mEq/L
4.2 mEq/L
24 mEq/L
12 mg/dL
1.0 mg/dL
100 mg/dL

## Slide 3
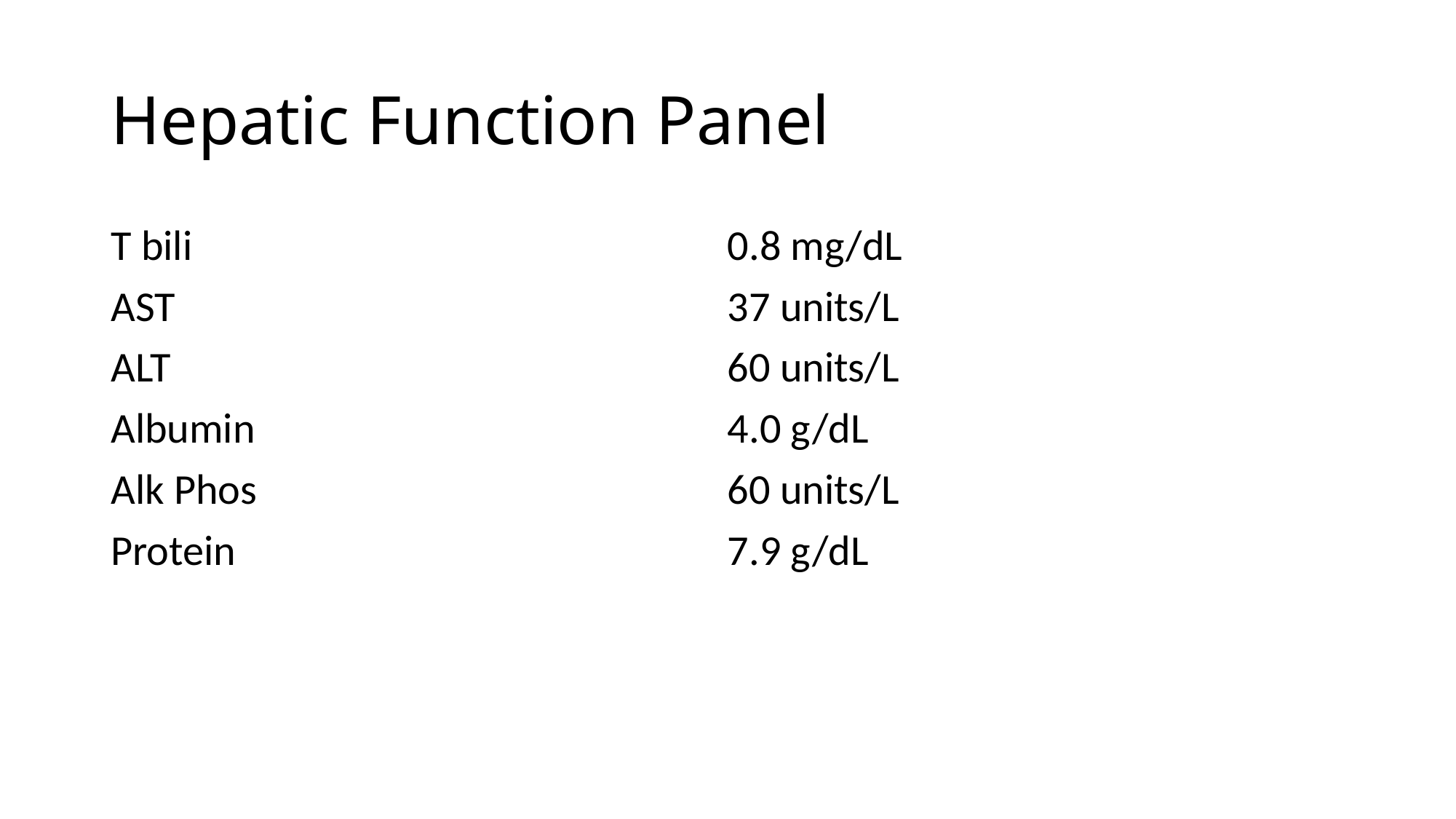

# Hepatic Function Panel
T bili
AST
ALT
Albumin
Alk Phos
Protein
0.8 mg/dL
37 units/L
60 units/L
4.0 g/dL
60 units/L
7.9 g/dL

## Slide 4
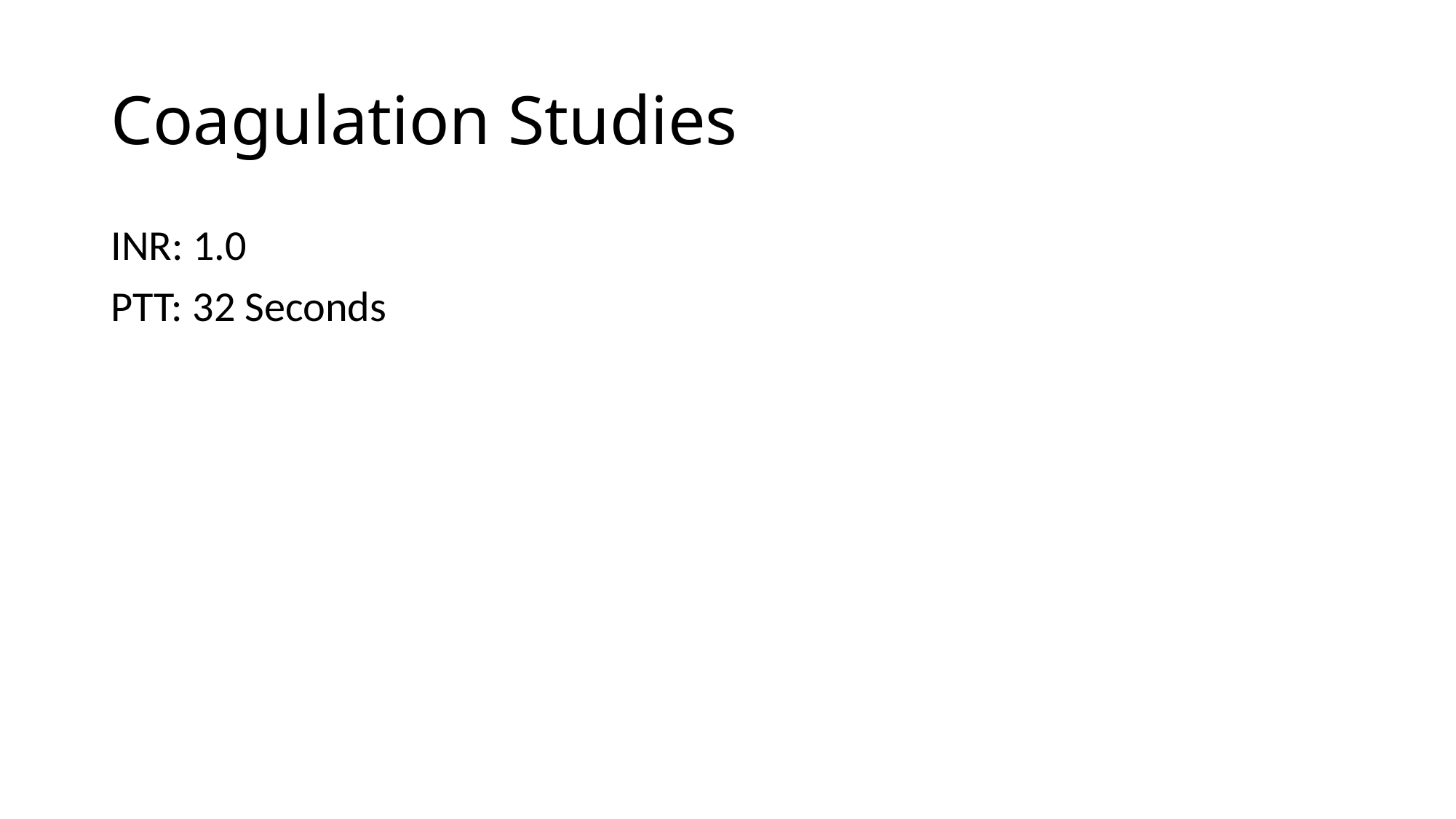

# Coagulation Studies
INR: 1.0
PTT: 32 Seconds

## Slide 5
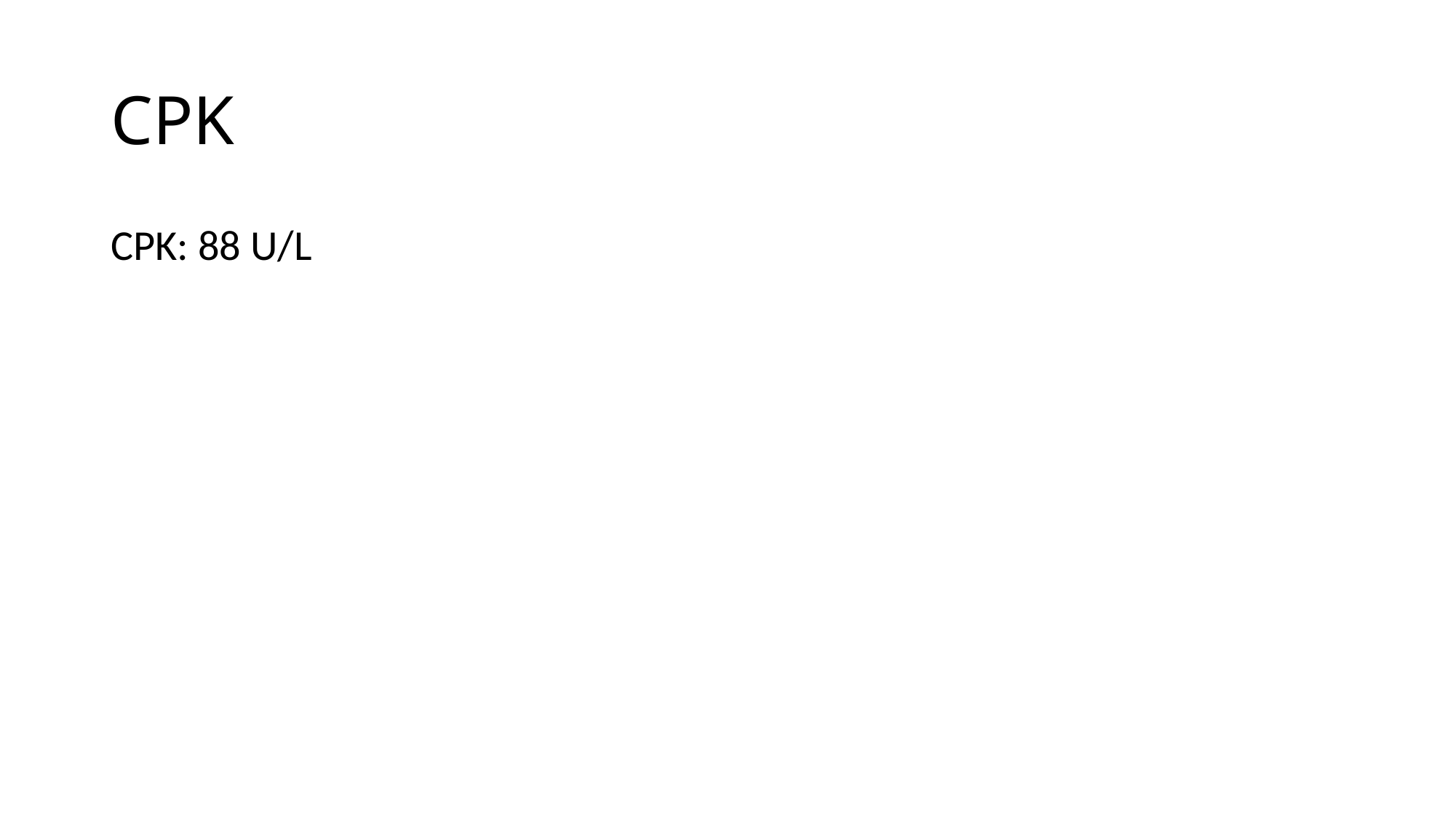

# CPK
CPK: 88 U/L

## Slide 6
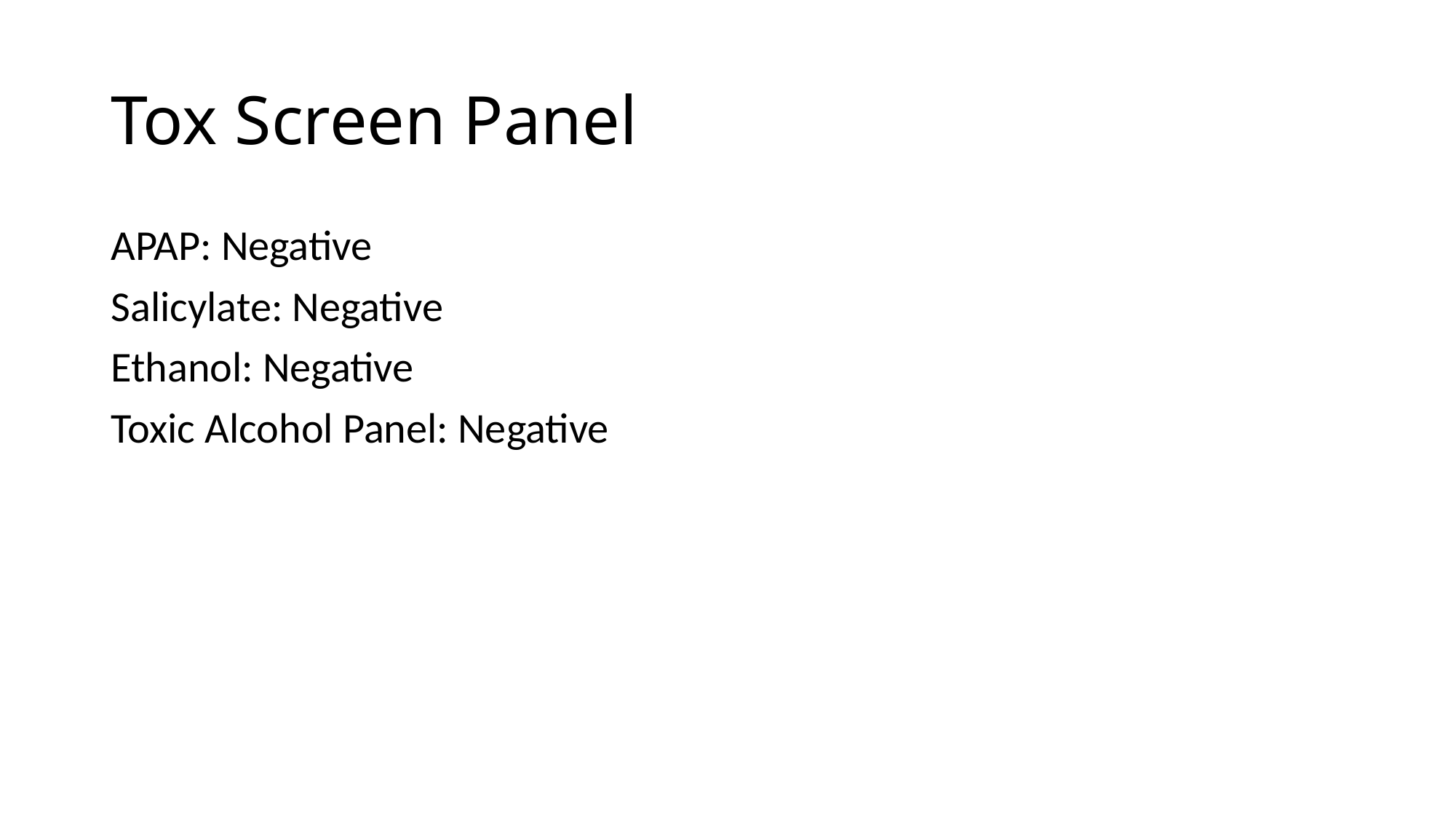

# Tox Screen Panel
APAP: Negative
Salicylate: Negative
Ethanol: Negative
Toxic Alcohol Panel: Negative

## Slide 7
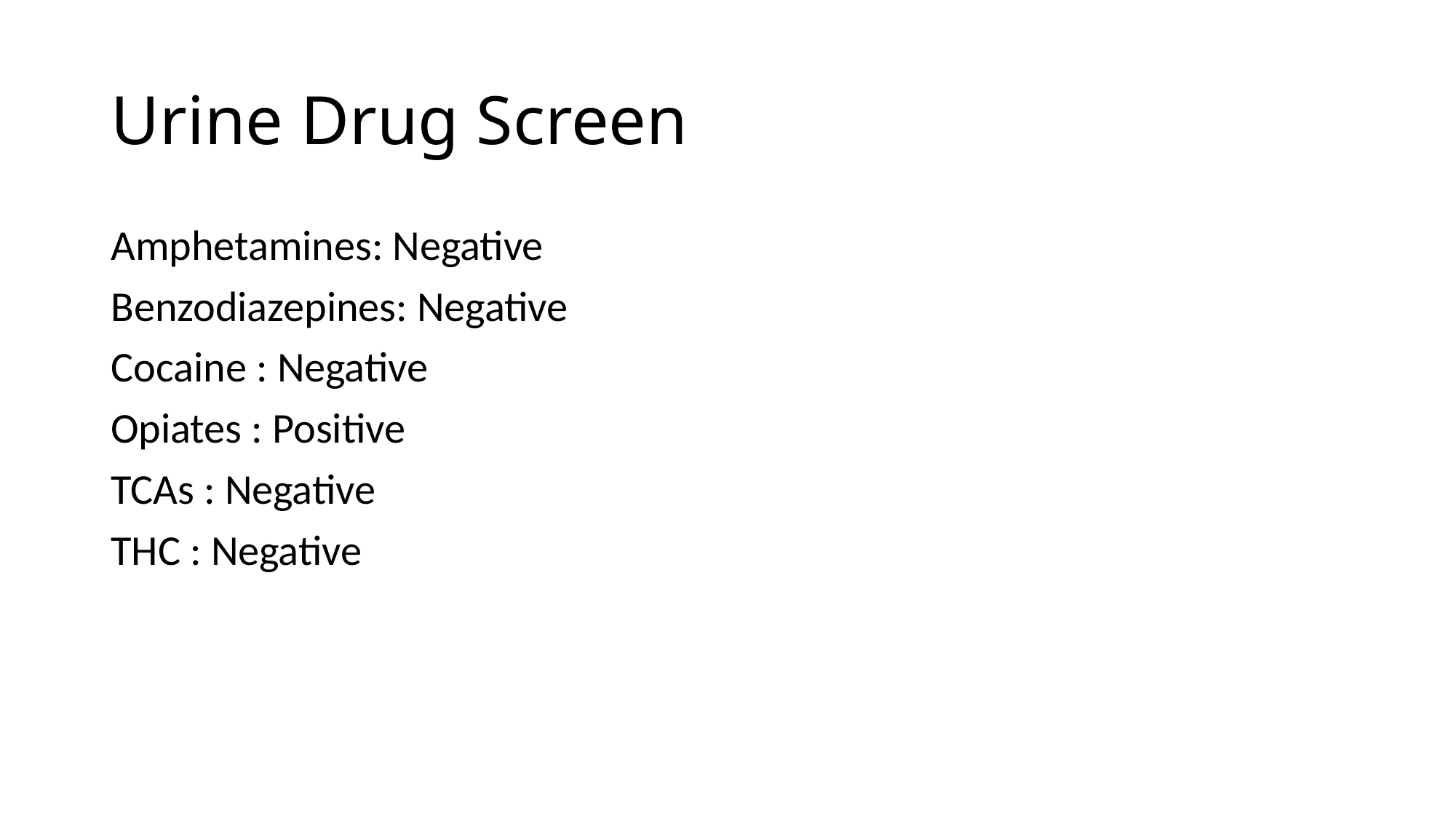

# Urine Drug Screen
Amphetamines: Negative
Benzodiazepines: Negative
Cocaine : Negative
Opiates : Positive
TCAs : Negative
THC : Negative

## Slide 8
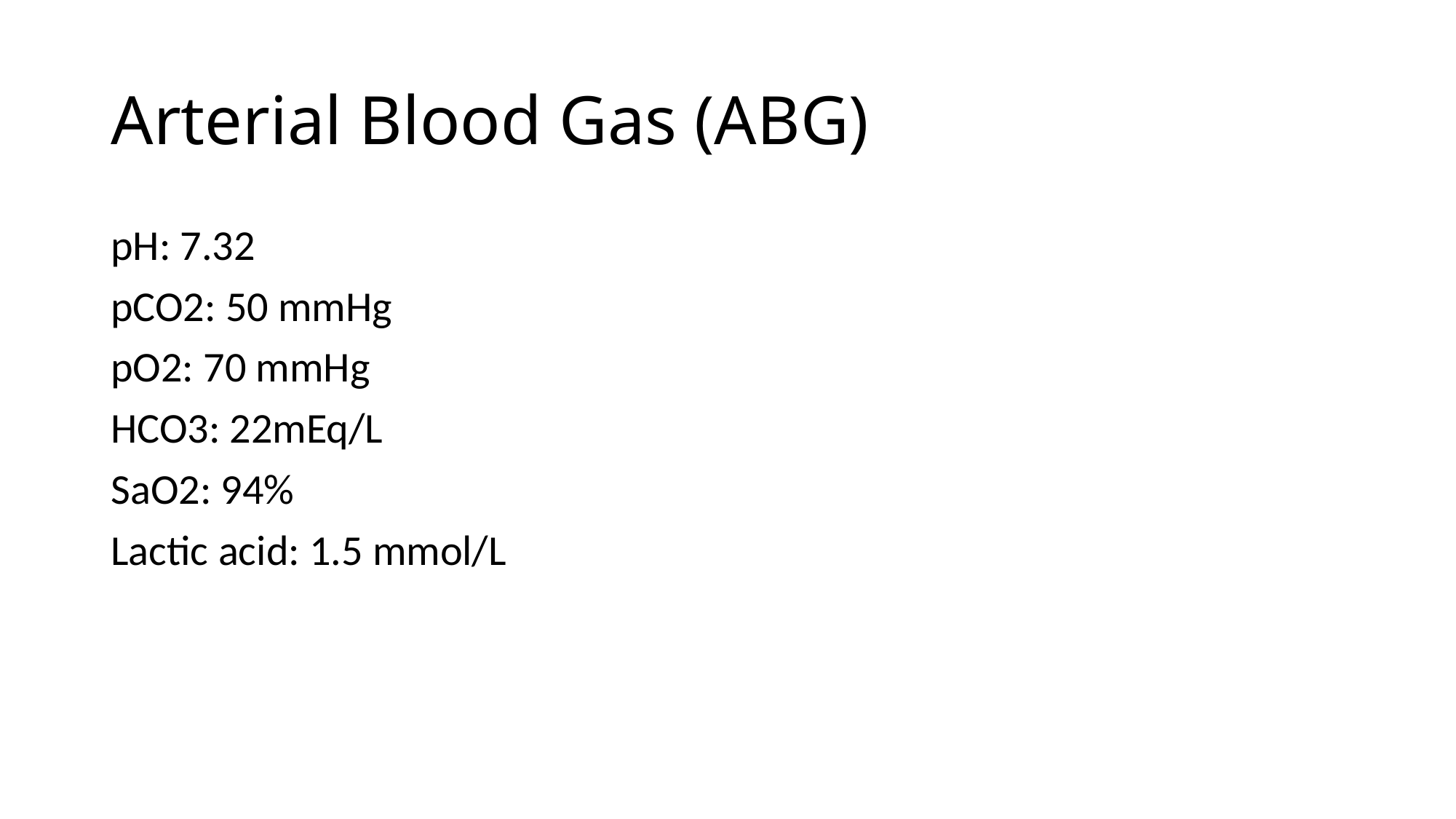

# Arterial Blood Gas (ABG)
pH: 7.32
pCO2: 50 mmHg
pO2: 70 mmHg
HCO3: 22mEq/L
SaO2: 94%
Lactic acid: 1.5 mmol/L

## Slide 9
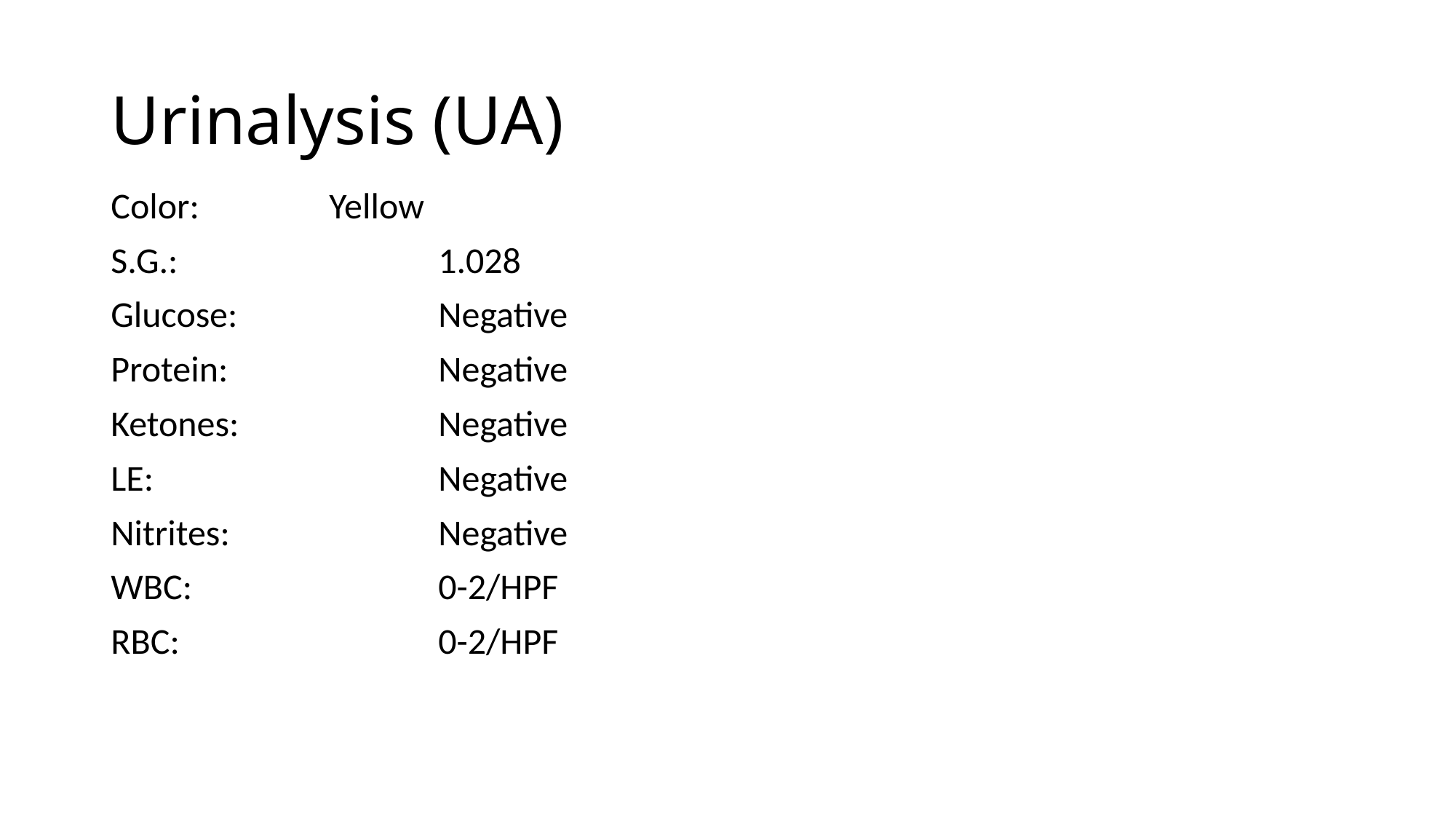

# Urinalysis (UA)
Color: 		Yellow
S.G.: 			1.028
Glucose:		Negative
Protein:		Negative
Ketones:		Negative
LE:			Negative
Nitrites:		Negative
WBC:			0-2/HPF
RBC: 			0-2/HPF

## Slide 10
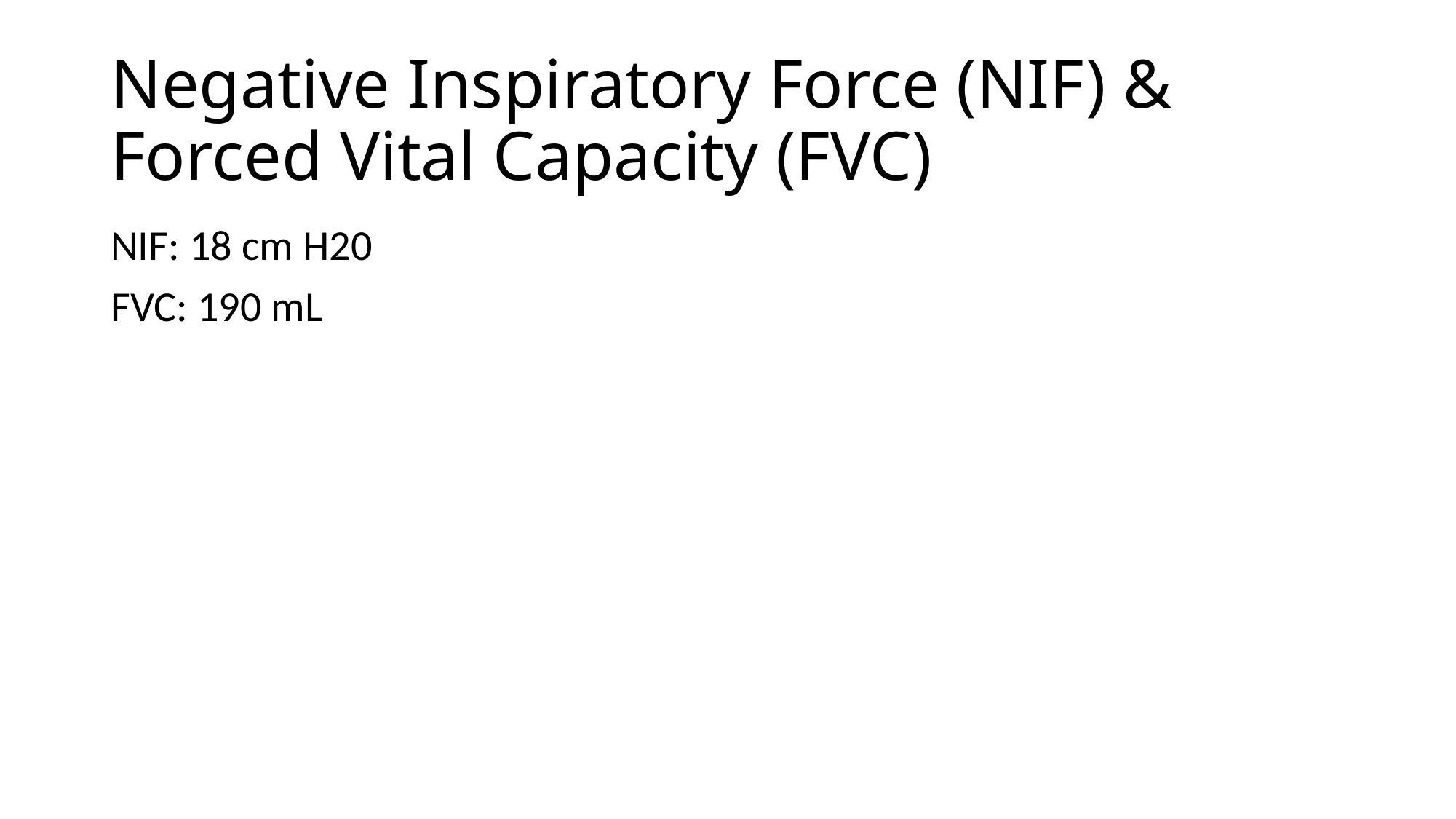

# Negative Inspiratory Force (NIF) & Forced Vital Capacity (FVC)
NIF: 18 cm H20
FVC: 190 mL

## Slide 11
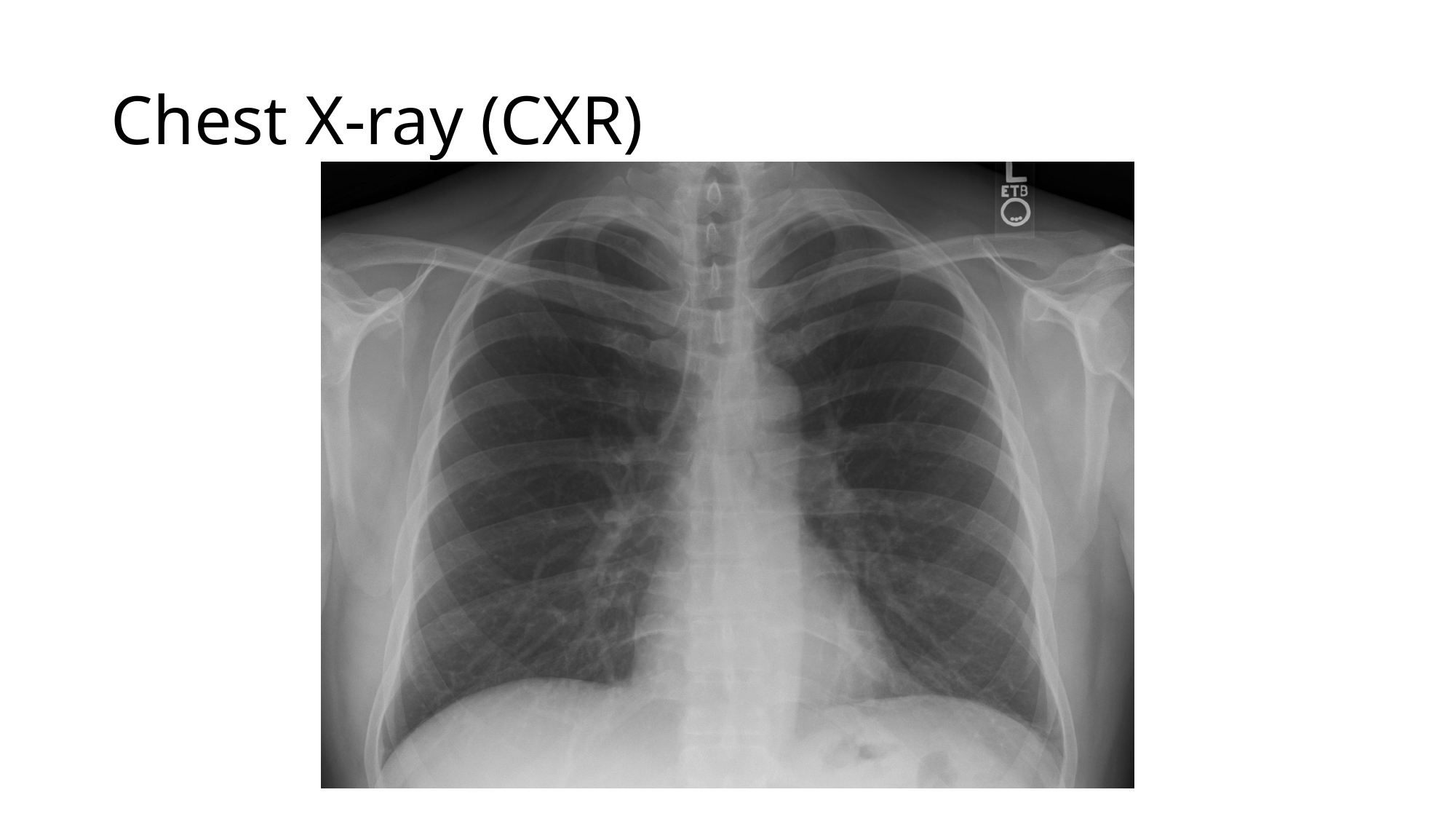

# Chest X-ray (CXR)

## Slide 12
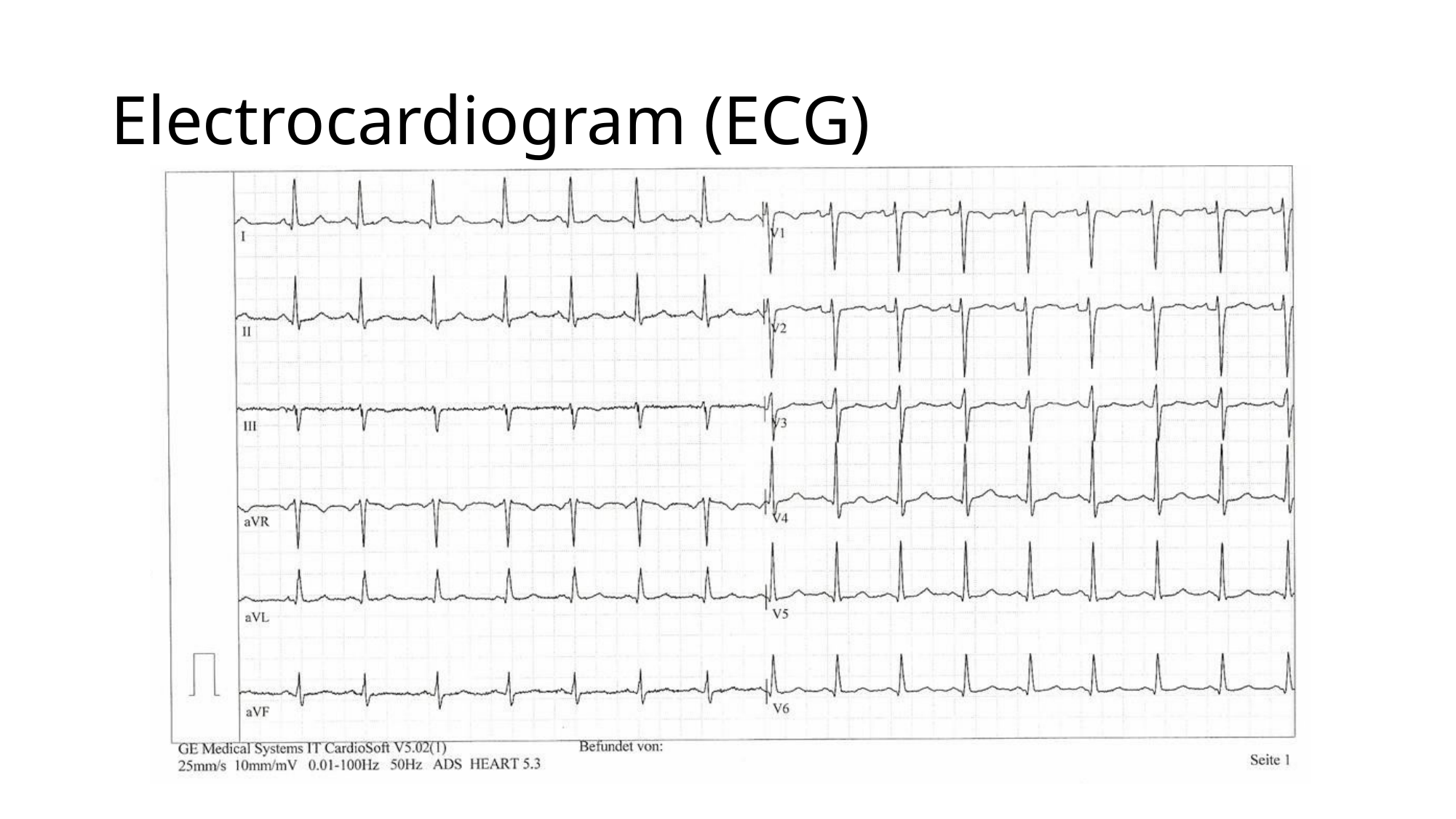

# Electrocardiogram (ECG)
